# Trading Tactics: Time to Rethink the Global Trade in Wildlife

**DOI:** 10.3390/ani10122456

**Published:** 2020-12-21

**Authors:** Neil D’Cruze, Jennah Green, Angie Elwin, Jan Schmidt-Burbach

**Affiliations:** World Animal Protection, 222 Gray’s Inn Rd., London WC1X 8HB, UK; JennahGreen@worldanimalprotection.org (J.G.); AngieElwin@worldanimalprotection.org (A.E.); JanSchmidt-Burbach@worldanimalprotection.org (J.S.-B.)

**Keywords:** animal welfare, conservation, COVID-19, pandemics, wildlife trade

## Abstract

**Simple Summary:**

The Covid-19 outbreak has brought about fresh and intensified scrutiny of the wildlife trade, which substantively involves commerce in exotic pets. In response, there have been calls for trade bans involving key components of the global commercial wildlife trade, and some major policy decisions involving trade bans have ensued. Yet, these actions have been criticised, largely based on concerns that they risk exacerbating poverty, undermining human rights, damaging conservation incentives, and otherwise harming sustainable development and conservation efforts. Instead, many critics propose improved regulation of the status quo, with the intention of nurturing a legal, sustainable, safe, humane, and equitable wildlife trade. Here, we provide a countering view that draws attention to: (1) why the risks presented by the wildlife trade (to animal welfare, biodiversity, public health, and financial security) are manifold, and cannot be treated with complacency; (2) why the goal of a legal, sustainable, safe, humane, and equitable wildlife trade is misleading and unachievable; and (3) why moving towards an end to the commercial trade in wildlife should be our ultimate and more ambitious goal. We hope to stimulate further discussion on this issue both within the sustainability research and policy domains, identifying a path towards consensus on how best to protect wildlife, people, and planet.

**Abstract:**

The Covid-19 outbreak has brought about fresh and intensified scrutiny of the wildlife trade, which substantively involves commerce in exotic pets. In response, major policy decisions involving trade bans have ensued, with calls for similar such action to be applied across the trade chain. Yet, these measures have been criticised, largely based on concerns that they risk exacerbating poverty, undermining human rights, damaging conservation incentives, and otherwise harming sustainable development and conservation efforts. Instead, many critics propose improved regulation of the status quo, with the intention of nurturing a legal, sustainable, safe, humane, and equitable wildlife trade. Herein, we provide a countering view that outlines how the risks presented by the wildlife trade are becoming increasingly recognised as being both manifold and severe; and raise concerns that the goal of a well-regulated wildlife trade is becoming increasingly exposed as a mirage. We conclude that while pursuing the United Nation’s Sustainable Development Goals (with their focus on poverty alleviation, food security, public health, and conservation) is enduringly vital, a flourishing wildlife trade is not. Given that the exploitation of wildlife, including for the pet trade, has been identified as one of the dominant drivers of biodiversity loss, emergence of zoonotic infectious disease, animal suffering, and financial instability, perpetuating the concept of utilising a regulated wildlife trade as the default approach to protect people and planet is in urgent need of re-evaluation.

## 1. Introduction

The Covid-19 outbreak, thought to have originated from the trade in wildlife [1,2,3], has brought about fresh and intensified scrutiny of this global phenomenon [2,4]. In response, major policy decisions involving wildlife trade bans have ensued; China has decided to ban the consumption of wild animals for food to safeguard people’s lives and health [5,6], and Vietnam has launched a new taskforce committed to reforming policies to prohibit the commercial trade and consumption of wild birds and mammals [7]. More widely, there have been calls for similar such action to be applied across the trade chain [8,9].

Yet, these calls for trade bans involving key components of the global commercial wildlife trade (including for exotic pets, in addition to luxury goods and food, entertainment, and traditional medicine) have been criticised as being neo-colonial and/or naïve, largely based on concerns that they risk exacerbating poverty, undermining human rights, damaging conservation incentives, and otherwise harming sustainable development and conservation efforts [10,11]. Instead, many critics propose improved regulation of the status quo, with the goal being a legal, sustainable, safe, humane, and equitable wildlife trade [10,12,13,14,15].

Herein, we outline a countering view that draws attention to: (1) why the risks presented by the wildlife trade (to animal welfare, biodiversity, public health, and financial security) are manifold, and cannot be treated with complacency; (2) why the goal of a legal, sustainable, safe, humane, and equitable commercial wildlife trade (being distinct from non-commercial trade such as animal rescue, conservation, and subsistence purposes) is misleading and unachievable; and (3) why moving towards an end to the commercial trade in wildlife should be our ultimate and more ambitious goal. We hope that we can stimulate further debate on this issue, identifying a path towards consensus on how best to protect wildlife, people, and planet.

## 2. Risks Presented by the Wildlife Trade Are Manifold, and Cannot Be Treated with Complacency

In light of recent evidence, there are growing concerns that the negative impacts of the wildlife trade are being ignored, down played, and treated with a lack of urgency.

### 2.1. Biodiversity Risks

The trade in wildlife for exotic pets, in addition to luxury goods and food, entertainment, and traditional medicine, is now so substantial that it represents one of the most prominent drivers of vertebrate extinction risk globally [16]. Trade of wild animals to meet the demands of growing local and global markets was also ranked among five key drivers of harmful ecosystem change in the most recent global assessment of biodiversity and ecosystem services [17]. Wildlife trade also puts entire ecosystems at risk by facilitating the introduction of species to new regions, where they can compete with (or outcompete) native species for resources and alter ecosystems [18], and contribute to biodiversity loss via pathogen emergence [19,20]. Furthermore, genetic pollution of wild populations, leading to the erasure of genetically distinct populations, can occur as part of “sustainable” captive breeding and trading processes if effective management is not in place [21].

### 2.2. Public Health Risks

Similarly, with regards to global public health, we again ignore the negative impacts of wildlife trade at our peril, as wild animal species are thought to be the source of at least 70% of all zoonotic emerging infectious diseases [22] and can provide opportunities for the inadvertent movement of pathogens across global boundaries [23]. Studies have warned that the stream of new emerging zoonotic diseases of public health concern is likely to continue at an ever-increasing rate under current conditions, calling the trade of wild animals a “perfect microbial storm” for pathogenic disease [24]. Furthermore, international wildlife trade brings infectious diseases to a global scale, amplifying the potential consequences of disease outbreaks and presenting a wider threat to more people (in addition to ecosystems and economies) than may otherwise have occurred if they were restricted to localised regions [23].

### 2.3. Animal Welfare Risks

Wildlife trade also bears substantial negative consequences for animal welfare because the potential for suffering (relating to both physical and mental domains) exists at each stage of the trade chain, including capture, captive breeding, transport, slaughter, and private ownership [25,26]. Too often, there is a failure to acknowledge that vertebrates (and some invertebrates) are generally considered sentient [27,28,29,30], which can lead to both extreme overt impacts, and more subtle chronic impacts on their well-being [28]. This oversight, in turn, exacerbates the aforementioned public health risk because wild animals often experience compromised immune systems when subject to debilitating captive conditions [31]. In combination with scenarios that allow for cross-species transmission (e.g., through close proximity to other species during transport or trade), the issue of a stress-related compromised health state can further amplify disease emergence risks [32].

### 2.4. Financial Security Risks

The wildlife trade can act as a valuable source of financial income for hunters, farmers, exporters, and vendors alike [33,34,35]. However, even if the intrinsic value of wildlife is discounted (i.e., the value that wildlife possesses in its own right, as opposed to the instrumental or “resource” value [36]), a painfully topical question is whether the income generated from the legal trade in wildlife (estimated to be worth billions of US dollars globally per year) offsets the full longer term economic costs of its operation. For example, when considering the financial impacts of zoonotic diseases alone, the monetary costs associated with pandemics (which potentially may be measured in trillions of dollars annually [37]) can far outweigh the financial benefits implied by the wildlife trade (which potentially may be measured in billions of dollars annually [38]). Furthermore, evidence suggests that too often only a relatively small proportion of the economic benefits of commercial wildlife trade reaches the poorest local communities [39], yet these beneficiaries (and other marginalised communities around the world [40]) are likely to bear the greatest economic burden and suffer the slowest economic recovery during fallout from a zoonotic disease outbreak [41,42].

## 3. The Goal of a Well-Regulated Wildlife Trade Is a Mirage

Fully evidenced case studies of sustainable, safe, and humane wildlife trade are the rarity rather than the norm, and there are growing concerns that, overall, the challenges involved are insurmountable.

### 3.1. Sustainability

A predominant approach adopted by some conservationists and policy makers has been the belief that sustainable use of wildlife is necessary to prevent biodiversity loss [43]. Here, the implication or hope appears to be that commodification and commercialisation enables nature to pay for its continued existence, whilst bringing benefits—both financial and social—particularly to those living in close proximity to wild populations [44]. However, there are increasing concerns that this status quo as a default approach is not tenable, particularly given that the systemic lack of scientific data on the status of wild populations, and ineffective management and monitoring of trade, impairs current sustainability efforts [45,46,47,48]. In particular, commonly applied “sustainable solutions”, such as commercial captive breeding and ranching of wild animals, are not always as sustainable as intended, given they may only be appropriate for a limited number of wild animal species that fit certain specific criteria [21,49,50].

### 3.2. Public Safety

Efforts to reduce the biosecurity risks associated with the global wildlife trade face substantial challenges. Risk of zoonotic disease transmission is inherent in every step of the wildlife trade chain, from source to final destination [51,52,53]. Although biosecurity protocols can help to lower the risk of zoonotic disease introduction, current surveillance systems for wildlife are universally inadequate for detection of clinical disease or pathogen presence [54,55,56]. Asymptomatic carriers and unidentified emerging pathogens can evade even highly sophisticated disease surveillance [57]. Infected animals can also go undetected because the large volumes of wildlife imported globally each year render it challenging and costly to effectively screen all individuals [55]. Furthermore, given the novel nature of emerging diseases, it is challenging to target surveillance to detect diseases that are not yet known.

### 3.3. Legality

When considering calls to improve wildlife trade regulation, it is critical to consider that legal and illegal trade are not always easily distinguishable, and a close complex relationship exists between these markets [58]. Wildlife trade can be legal, illegal, or a combination of both, depending on how a species is classified as it moves throughout the market chain [59]. Legal wildlife trade can also be difficult to monitor due to unintentional mistakes, such as inadequate record keeping [60,61], and mislabelling of species [59]. This creates opportunity for crossover and intentional fraudulent activity, such as when legal operations, including wildlife farms, act as “cover” to launder poached wildlife [62]. Similarly, criminal networks are known to seek influence over legally operating wildlife industries [49], and previous attempts to sustainably manage some aspects of legal trade have failed due to their involvement [63].

### 3.4. Humane Trade

Global understanding, attitudes, and ethical standards are evolving to the extent that, in some markets, the negative impact on the lives of individual wild animals being exploited commercially is becoming increasingly socially and culturally unacceptable [64]. However, currently there is no overarching body to regulate or address the impacts of the global supply of wildlife on animal welfare [65,66], and although a number of international entities and corporations could play an influential role, it is not always immediately clear where responsibility lies [66,67]. A fundamental question is (again when placing the intrinsic value [36] of wild animals aside) whether the conditions of the wildlife trade can be improved, from source to final destination, to a degree that enables wild animals to thrive, rather than merely survive, during the trade chain whilst still generating a financial profit.

### 3.5. Equitability

It is important to note that wildlife trade can cause environmental injustice that burdens the very same communities who rely on wildlife for livelihoods [16]. Indigenous peoples and local communities are facing growing threats from resource extraction, commodity production, mining, and transport and energy infrastructure, with various consequences for local livelihoods and health [17]. Among the local indicators developed and used by indigenous peoples and local communities, 72% show negative trends in nature that underpin local livelihoods and well-being [17]. Data show some communities perceive harvesting wildlife for export as a sporadic, unreliable, and risky source of income [68], and case reports of infectious diseases demonstrate that direct interaction with wild animals for farm workers can place them at a heightened risk of zoonotic disease transmission [69]. In addition, in some cases, wages are so low they manage only to keep families above the extreme poverty line [69].

## 4. Moving Towards an End to the Wildlife Trade, the Case for a More Ambitious Goal

Caution must be taken to ensure that our ultimate goal remains a safe, sustainable, and humane planet, rather than an economically robust commercial wildlife trade.

### 4.1. Aiming High

Wildlife trade is an immense and multifaceted industry that involves a vast array of animals (in addition to plants and fungi) [70]. Although some of these transactions represent luxury products for the world’s elites [71], others have nutritional and medicinal significance for some of the world’s most vulnerable people, especially in developing countries [58]. For others, wildlife serves as casual captive ornaments, status symbols, or exotic pets. Yet it is the United Nation’s Sustainable Development Goals (with their focus that includes poverty alleviation, food security, public health, and conservation [72]) that are enduringly vital and must be pursued, not a booming wildlife trade. Given that the exploitation of wildlife has been identified as one of the dominant drivers of biodiversity loss, emergence of zoonotic infectious disease, animal suffering, and financial instability [16,73,74], perpetuating the concept of utilising a regulated wildlife trade as default approach to protect people and planet is in urgent need of re-evaluation.

### 4.2. Benefits of Bans

Specifically, trade bans apply a more cautionary approach that effectively removes the current onus from the conservationist to prove trade is unsustainable [75] (which may come too late—if at all—to prevent associated extinctions and loss of income) [76], removes opportunities for legal trade to operate as a cover for illegal activity [77], maximises the chances of preventing the spread and emergence of zoonotic disease [78], reduces species invasion risk [79], and ameliorates current or removes future negative animal welfare impacts incurred throughout [25]. In addition, there is also increasing evidence that the punitive consequences of illegal trade may be far more likely to change consumer attitudes towards their intention to purchase wildlife, rather than discouragement focused on the negative impacts such behaviour would have on animal welfare and conservation [80]. Irrespective of the rationale for why they have been established, the reality is that wildlife trade bans exist at a local, provincial, federal, national, and international level across the globe, and are relied upon in scenarios where the negative impacts have been deemed unacceptable by the public and legislators (as is true of legislation pertaining to other illegal activities in society in addition to wildlife trade) [81].

### 4.3. Bans in Practice

Although the effectiveness of wildlife trade bans has been contested (e.g., [10,11,82]), there are numerous examples of bans in current operation that demonstrate their practical value. For instance, in terms of conservation benefits, overall the EU ban on imports of wild-caught birds in 2005 is thought to have effectively reduced trade and biological invasion risk globally [79,83]. Similarly, Pain et al. [84] reported that the US ban on imports of birds (on the Convention on International Trade in Endangered Species of Wild Fauna and Flora (CITES) Appendices I and II) has had a positive impact on parrot conservation in the Neotropics. Furthermore, national bans (on taking birds from nests for the international pet trade) are also considered to have significantly increased parrot nest success across diverse geographical locations and political-economic conditions (including in Africa, Asia, and Australia) [84]. Therefore, it is perhaps unsurprising that many non-governmental organisations (NGOs) and academic researchers have also recommended commercial wildlife trade bans as a preferred conservation measure in relation to a range of taxa across a variety of trade scenarios (e.g., [48,85,86,87]). For example, Ferretti et al. [88] projected that a blanket ban on shark fin trade in the US would have a considerable positive conservation impact, Marshall et al. [48] proposed a ban on the international reptile trade to reduce the pressures on wild populations, and, more broadly, Frank and Wilcove [47] warned that the lack of bans on international wildlife trade poses a serious threat to species extinction.

### 4.4. Challenges of Bans

Effective application of trade bans is not without its challenges, and caution must be taken to avoid any unintended negative impacts. However, arguably, trade bans are no different to other efforts towards improving the wildlife trade in this regard. In particular, in order to prevent the rerouting of legal trade flows, trade bans should be global and incorporate all sourcing methods, including animals that have been wild caught or otherwise sourced from commercial captive breeding facilities [78]. To prevent any subsequent illegal trade activity or opportunities for corruption [85], wildlife trade bans should also be accompanied with effective enforcement (including appropriate sentences and the political will to implement them) [89], in addition to well-designed mass media campaigns to reduce consumer demand, and otherwise secure public support [80]. To prevent any damaging impacts on wildlife protection efforts (including potential for intentional killing of wild animals due to human–wildlife conflicts [90]), human development goals, or human rights [10], collaborative arrangement that gives agency to local communities and stakeholders while incorporating global perspectives will enable a multi-faceted and versatile approach [91], for example, a phased shift away from a financial dependence on the wildlife trade.

## 5. Conclusions

A re-evaluation of the current modus operandi for the global wildlife trade is urgently required. In response to COVID-19 (and increasing recognition of the other severe threats presented by the global wildlife trade), it is now critical for the global community to re-consider whether the concept of utilising a regulated commercial wildlife trade (being distinct from non-commercial trade such as animal rescue, conservation, and subsistence purposes) as a default mainstream approach to protect people and planet remains a wise endeavour to be actively pursued. Together, we must ensure that our ultimate goal is a healthy civilisation that is in harmony with, and engages responsibly within, our planetary boundaries. Yet, this goal will be no easy task. Rather, as with efforts to tackle other global challenges, such as climate change, it will require bold and progressive thinking, along with the fortitude to engage proactively with all stakeholders across all levels of engagement, including those who are profiting financially, or otherwise resistant to consider a shift away from the detrimental aspects of the status quo.
